# Prevalence and Correlates of Osteoporosis and Metabolic Syndrome Among Patients With Chronic Obstructive Pulmonary Disease at a Rural Tertiary Healthcare Center in India

**DOI:** 10.7759/cureus.78759

**Published:** 2025-02-09

**Authors:** Ruchira Roy, Adesh Kumar

**Affiliations:** 1 Department of Respiratory Medicine, Uttar Pradesh University of Medical Sciences, Etawah, IND

**Keywords:** bmd, bmi, dexa scan, hdl-c, metabolic syndrome, ncep-atpiii, osteopenia, osteoporosis, t-score, who

## Abstract

Background

Chronic obstructive pulmonary disease (COPD) results from chronic inflammation triggered by various risk factors. This inflammation can also impact other organ systems. COPD patients often have comorbidities such as osteoporosis and metabolic syndrome. Osteoporosis is a skeletal disorder characterized by reduced bone mineral density (BMD), while metabolic syndrome encompasses central obesity, hypertriglyceridemia, low high-density lipoprotein cholesterol, hyperglycemia, and hypertension. The coexistence of both osteoporosis and metabolic syndrome in COPD patients has not been previously studied in India.

Aim and objectives

The aim of this study is to determine the prevalence of osteoporosis and metabolic syndrome, as well as their associated factors, among patients with COPD at a rural tertiary healthcare center in India.

Materials and methods

A total of 363 COPD patients were included in this study, comprising 153 males and 210 females. This hospital-based cross-sectional study was conducted at a rural tertiary healthcare center in India. The diagnosis of COPD and classification of airflow limitation severity were based on the Global Initiative for Chronic Obstructive Lung Disease (GOLD) 2020 guidelines. The WHO criteria for osteoporosis and osteopenia were used to diagnose osteoporosis in the study participants. BMD was measured using dual-energy X-ray absorptiometry (DEXA). Metabolic syndrome was diagnosed using the National Cholesterol Education Program: Adult Treatment Panel III criteria.

Results

The mean age of patients diagnosed with osteoporosis was 68.42 ± 11.38 years, while the mean age of those diagnosed with metabolic syndrome was 64.46 ± 10.37 years. Among the 363 study participants, the prevalence of osteoporosis was 70.25%, and the prevalence of metabolic syndrome was 62.53%. A significant association was found between the GOLD severity grading of COPD, BMI, education level, and socioeconomic status with the T-score of the DEXA scan. However, no significant association was observed between the duration and route of corticosteroid administration and the T-score categories of BMD. In contrast, metabolic syndrome was significantly associated with GOLD severity grading, BMI, education level, socioeconomic status, duration and route of corticosteroid administration, smoking status, and duration of biomass fuel exposure. Additionally, smoking status and biomass fuel exposure duration also showed a significant association with osteoporosis. Notably, metabolic syndrome itself was significantly associated with the presence of osteoporosis.

Conclusions

Both osteoporosis and metabolic syndrome are highly prevalent among COPD patients in the Indian population. Several factors significantly influence the occurrence of these conditions, including the severity of COPD, BMI, education level, socioeconomic status, smoking status, and duration of biomass fuel exposure. Furthermore, metabolic syndrome itself plays a crucial role in the development of osteoporosis in individuals with COPD.

## Introduction

Chronic obstructive pulmonary disease (COPD) is a heterogeneous lung disorder characterized by persistent respiratory symptoms such as dyspnea, cough, sputum production, and exacerbations. These symptoms arise due to abnormalities in the airways (bronchitis and bronchiolitis) and/or alveoli (emphysema), leading to progressive airflow obstruction [1]. COPD is a major global cause of morbidity and mortality, imposing a substantial and growing economic and social burden [2].

COPD frequently coexists with other diseases (comorbidities) that significantly impact prognosis [3]. Metabolic syndrome, with an estimated prevalence of over 30% in COPD patients [4], is characterized by central obesity, hypertriglyceridemia, low high-density lipoprotein cholesterol (HDL-C), hyperglycemia, and hypertension [5]. Another common but often underdiagnosed comorbidity is osteoporosis, which is associated with poor health outcomes and prognosis [6]. Osteoporosis in COPD patients is linked to emphysema, low BMI, and reduced fat-free mass. Even after accounting for factors such as steroid use, age, smoking history, and exacerbations, COPD patients remain at an increased risk of low bone mineral density (BMD) and fractures [7].

## Materials and methods

This hospital-based cross-sectional study was conducted from January 2019 to August 2022 at a rural tertiary healthcare center in India. Ethical clearance was obtained from the Institutional Ethical Committee (approval number 170/2018). The study included patients who presented with a history, symptoms, signs, chest X-ray findings, and pulmonary function test values suggestive of COPD, as per the Global Initiative for Chronic Obstructive Lung Disease (GOLD) [8] guidelines, and who met the inclusion criteria. A total of 363 COPD patients were enrolled.

Patients included in the study were hemodynamically and mentally stable and provided informed consent. Exclusion criteria comprised patients with a recent myocardial infarction, other superimposed lung diseases, ventilator support, hemodynamic instability, pregnancy, or refusal to participate.

A predesigned questionnaire was used to collect data, including sociodemographic details such as age, gender, address, occupation, family income, educational qualification, and socioeconomic status. Additional data on exposure to biomass fuel (type and duration) and tobacco use (type, duration, and current smoking status) were recorded. Diagnoses were documented in clinical profile sheets, and pulmonary function test reports were collected to classify airflow limitation severity using GOLD grading [8].

Anthropometric measurements, including height, weight, and waist circumference, were recorded. Blood pressure was measured according to the American Heart Association’s recommendations, with readings taken from both arms in a supine position after a 15-minute rest period; the highest measurement was recorded. BMI was calculated as weight (kg) divided by height (m²) and categorized as underweight (<18.5 kg/m²), normal weight (18.5-24.9 kg/m²), overweight (25-29.9 kg/m²), or obese (>30 kg/m²) [9]. Waist circumference was measured using a measuring tape placed horizontally at the midpoint between the iliac crest and the lower border of the lowest rib, with patients standing upright and in light clothing. Measurements were taken at the end of normal expiration [5].

Routine and specialized blood investigations included a complete blood count, liver and kidney function tests, serum electrolytes, thyroid function tests, serum triglycerides, serum HDL-C, fasting blood sugar (FBS), and serum total calcium levels. To rule out other lung diseases and confirm COPD diagnoses, a PA chest X-ray was performed.

All patients underwent a dual-energy X-ray absorptiometry (DEXA) scan to measure BMD, specifically at the femoral neck, expressed in absolute terms as grams of mineral per square centimeter. Additionally, a lateral spine X-ray was performed to assess vertebral fractures and fracture history. The WHO classification for osteoporosis and osteopenia was used to categorize study subjects based on DEXA T-scores, which indicate the number of SDs above or below the reference value for a healthy young individual. According to WHO thresholds, a normal BMD is within 1 SD of the young adult reference mean (T-score ≥ -1.0), osteopenia is defined as a T-score between -1.0 and -2.5, osteoporosis is diagnosed when the T-score is ≤ -2.5, and severe (established) osteoporosis is characterized by a T-score ≤ -2.5 along with the presence of one or more fragility fractures [10].

The diagnosis of metabolic syndrome was based on the modified National Cholesterol Education Program: Adult Treatment Panel III criteria for Asians. According to these criteria, metabolic syndrome is defined as the presence of any three out of five risk factors: central obesity, indicated by a waist circumference of ≥90 cm (≥35 inches) for males and ≥80 cm (≥31 inches) for females; reduced HDL cholesterol, defined as <40 mg/dL (1.03 mmol/L) for males and <50 mg/dL (1.3 mmol/L) for females or being on drug treatment for low HDL-C; elevated serum triglycerides, defined as ≥150 mg/dL (1.7 mmol/L) or being on drug treatment for high triglycerides; elevated blood pressure, defined as systolic blood pressure ≥130 mmHg or diastolic blood pressure ≥85 mmHg or being on antihypertensive medication; and elevated fasting blood glucose, defined as ≥100 mg/dL or being on drug treatment for high blood glucose [5].

Data analysis

Data analysis was performed using IBM SPSS Statistics for Windows, Version 25.0 (Released 2017; IBM Corp., Armonk, NY, USA). Quantitative variables were summarized using mean, SD, median, range, minimum, and maximum values, while qualitative variables were presented as frequencies and proportions. The chi-square test was employed to assess associations between categorical variables, and Student’s unpaired t-test was used to evaluate differences in the mean values of various parameters related to metabolic syndrome and osteoporosis.

## Results

**Figure 1:**
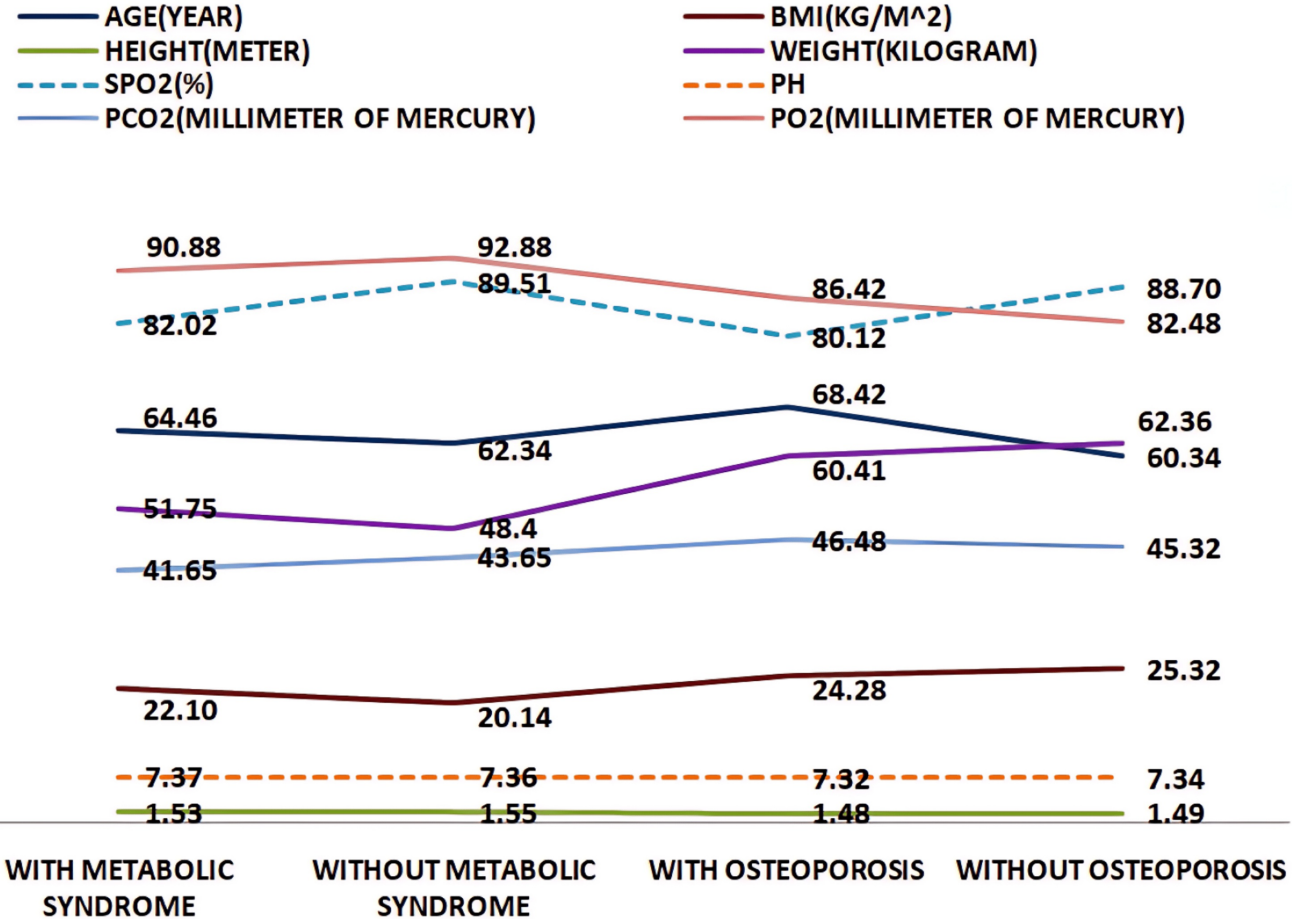
Line diagram depicting a comparison of mean values of age, BMI, height, body weight, SpO₂, pH, pCO₂, and pO₂ between patients with and without metabolic syndrome and patients with and without osteoporosis pCO₂, partial pressure of carbon dioxide; pH, potential of hydrogen; pO₂, partial pressure of oxygen; SpO₂, peripheral arterial oxygen saturation

A total of 363 COPD patients were included in this study. The mean age of patients diagnosed with osteoporosis was 68.42 ± 11.38 years, while those diagnosed with metabolic syndrome had a mean age of 64.46 ± 10.37 years. The mean BMI values for patients with osteoporosis and metabolic syndrome were 22.10 ± 4.38 kg/m² and 24.28 ± 5.34 kg/m², respectively. Patients with metabolic syndrome had higher mean body weight and BMI than those without metabolic syndrome. Additionally, their mean partial pressure of oxygen (pO₂) and peripheral arterial oxygen saturation (SpO₂) levels were lower compared to those without metabolic syndrome. In patients with osteoporosis, mean values of body weight, height, BMI, T-score of BMD, SpO₂, and potential of hydrogen (pH) were lower than in those without osteoporosis (Figure 1).

**Figure 2:**
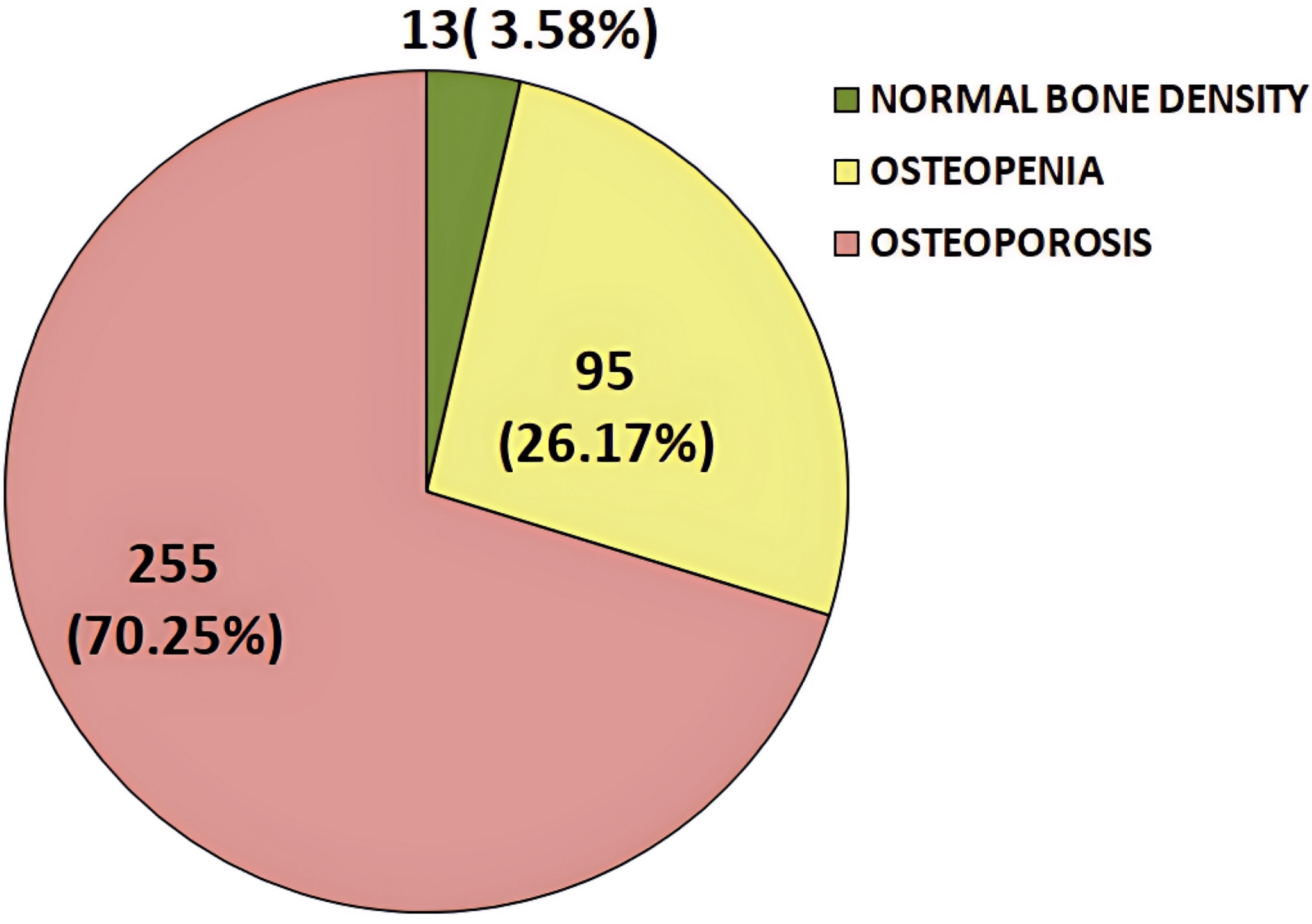
Pie chart illustrating the frequency distribution of study subjects with osteoporosis, osteopenia, and normal bone density based on T-score values from the DEXA scan, as per WHO criteria DEXA, dual-energy X-ray absorptiometry

In this study, among the 363 patients, 255 (70.25%) had a T-score of ≤-2.5, indicating osteoporosis, while 95 (26.17%) had a T-score between -1.0 and -2.5, classified as osteopenia. Only 13 (3.58%) patients had a T-score of -1.0 or above, considered normal. Thus, the prevalence of osteoporosis in the study population was 70.25%, based on the WHO criteria for osteoporosis diagnosis (Figure 2).

**Figure 3:**
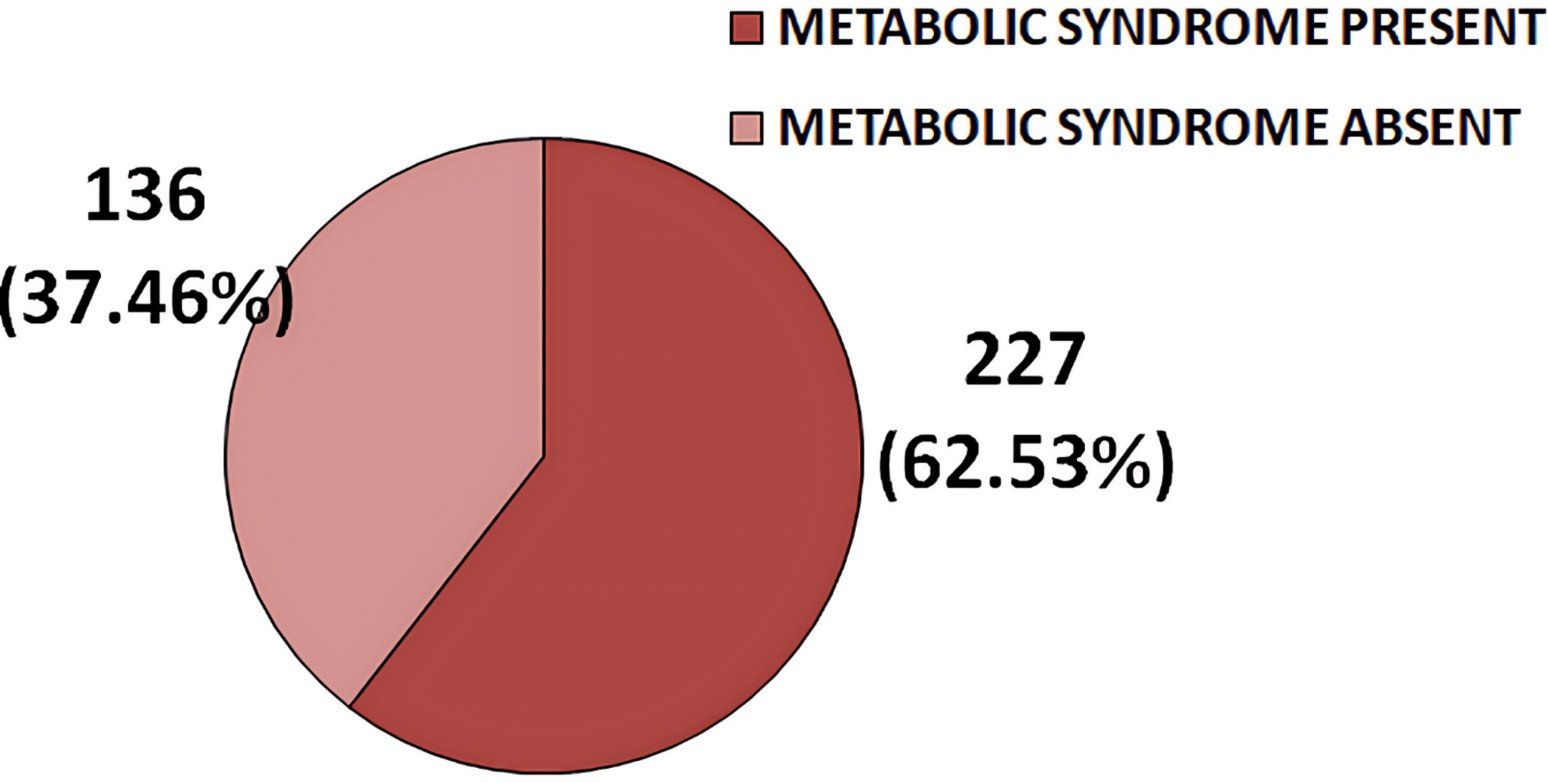
Pie diagram illustrating the frequency distribution of metabolic syndrome among study subjects based on NCEP-ATP III criteria NCEP: ATP III, National Cholesterol Education Program: Adult Treatment Panel III

In this study, 227 (62.53%) patients were diagnosed with metabolic syndrome based on the NCEP-ATP III criteria (Figure 3).

**Figure 4:**
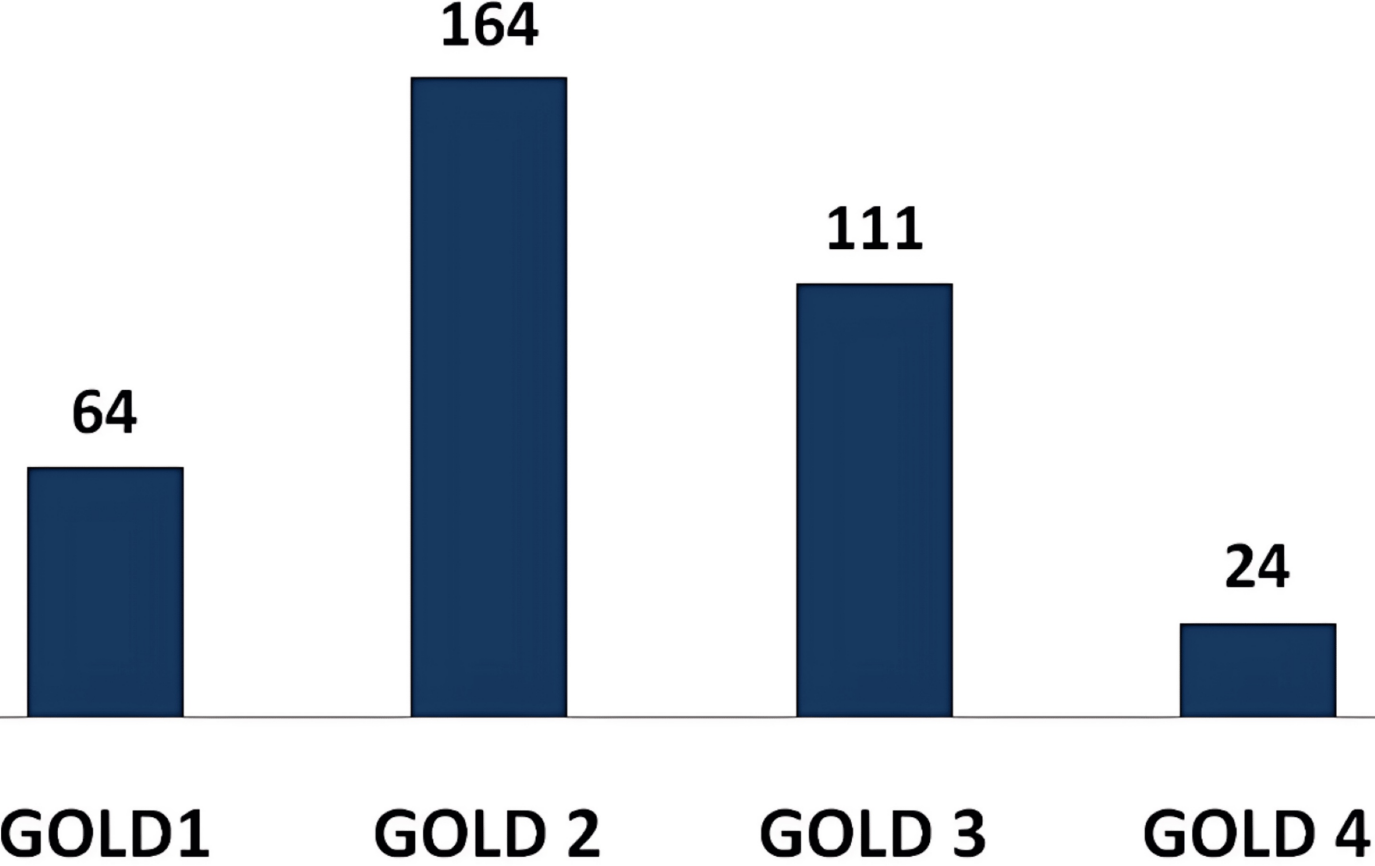
Bar diagram illustrating the frequency distribution of study subjects based on GOLD grading of airflow limitation GOLD, Global Initiative for Chronic Obstructive Lung Disease

Among the 363 COPD patients, the majority (164 patients) were diagnosed with GOLD grade 2 airflow limitation, followed by 111 patients with GOLD grade 3, and 64 patients with GOLD grade 1. The fewest patients, 24 in total, were diagnosed with GOLD grade 4 airflow limitation (Figure 4).

**Figure 5:**
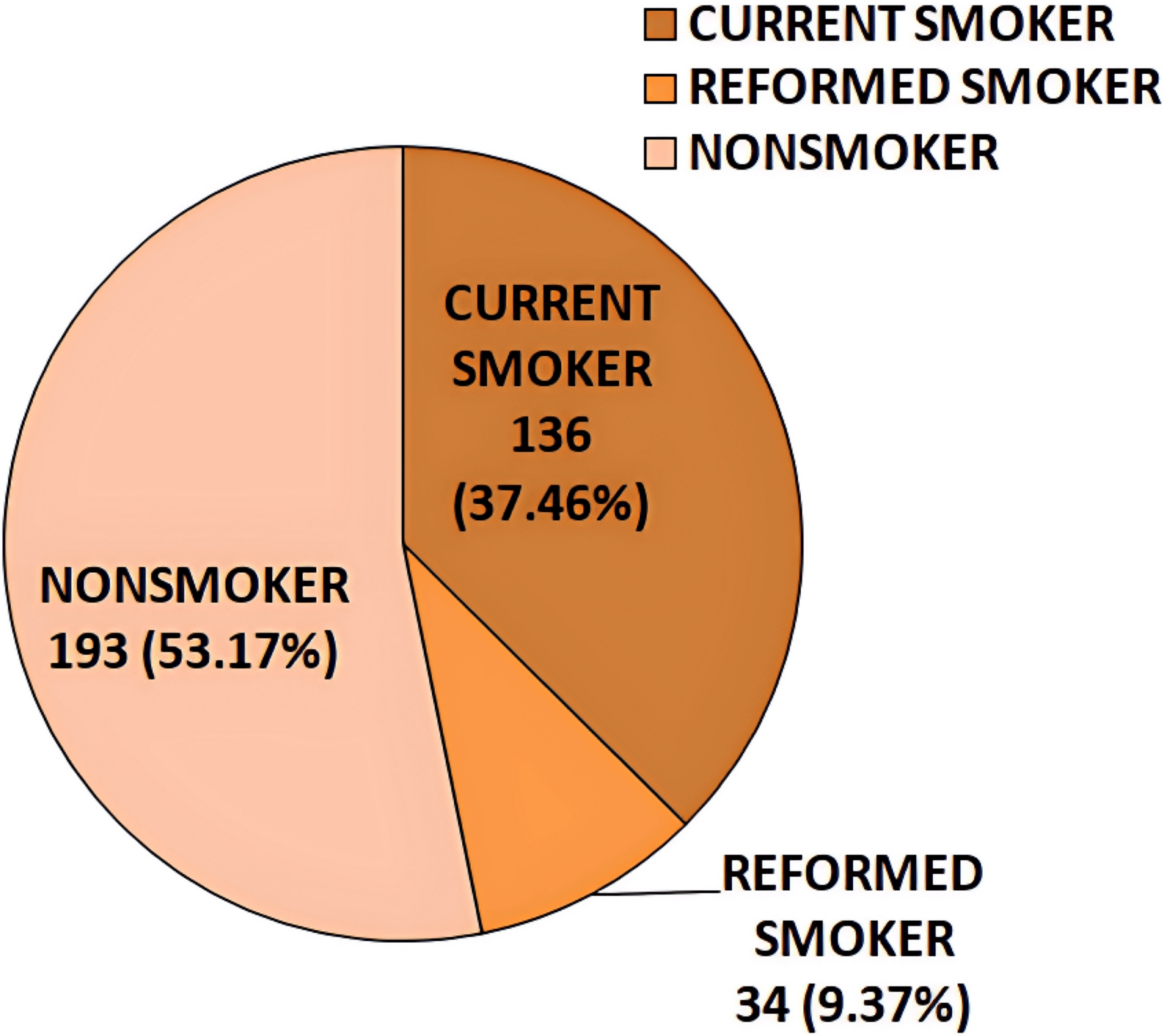
Pie diagram illustrating the frequency distribution of study subjects based on smoking status

In this study, out of 363 patients, 193 (53.17%) were nonsmokers, 136 (37.46%) were current smokers, and 34 (9.37%) were former smokers (Figure 5).

In this study, a significant association was observed between the severity of airflow limitation (p = 0.001), BMI (p < 0.001), education status (p = 0.01), and socioeconomic status (p = 0.02) with the T-score of the DEXA scan. However, the route of administration and duration of corticosteroid use showed no significant association with the T-score of the DEXA scan (Table 1).

**Table 1:** Association between GOLD grading of airflow limitation severity of COPD, BMI, route of administration of corticosteroid, duration of corticosteroid used, education status, and socioeconomic status of the patients versus T-score classification of BMD in DEXA scan ^*^ Statistically significant For statistical analysis, Pearson’s chi-square test was employed, with a p-value of less than 0.05 considered statistically significant. BMD, bone mineral density; COPD, chronic obstructive pulmonary disease; DEXA, dual-energy X-ray absorptiometry; GOLD, Global Initiative for Chronic Obstructive Lung Disease

Variable	Status	T-Score (n, %)			Total (n, %)	Statistical interpretation (chi-square test)
		≥ -1.0	-1.0 to -2.5	≤-2.5		
GOLD grading of airflow limitation severity	GOLD 1	6 (9.4%)	1 (25.0%)	42 (65.6%)	64 (17.6%)	p = 0.001^*^
	GOLD 2	5 (3.0%)	54 (32.9%)	105 (64.0%)	164 (45.2%)	
	GOLD 3	0 (0.0%)	19 (17.1%)	92 (82.9%)	111 (30.6%)	
	GOLD 4	2 (8.3%)	6 (25.0%)	16 (66.7%)	24 (6.6%)	
BMI of patients	Underweight	0 (0.0%)	12 (18.2%)	54 (81.8%)	66 (18.2%)	p < 0.001^*^
	Normal weight	2 (1.0%)	64 (31.2%)	139 (67.8%)	205 (56.5%)	
	Overweight	9 (10.8%)	16 (19.3%)	58 (69.9%)	83 (22.9%)	
	Obese	2 (22.2%)	3 (33.3%)	4 (44.4%)	9 (2.5%)	
Route of administration of corticosteroid	Not used	4 (6.34%)	20 (31.74%)	39 (61.90%)	63 (17.35%)	p = 0.438
	Oral	3 (2.65%)	26 (23.0%)	84 (74.33%)	113 (31.12%)	
	Inhaled	6 (3.20%)	49 (26.20%)	132 (70.58%)	187 (51.52%)	
Duration of corticosteroid used	Not used	4 (6.34%)	20 (31.74%)	39 (61.90%)	63 (17.35%)	p = 0.502
	<1 year	6 (3.15%)	47 (24.73%)	137 (72.10%)	190 (52.34%)	
	≥1 year	3 (2.72%)	28 (25.45%)	79 (71.81%)	110 (30.30%)	
Education status	Illiterate	0 (0.0%)	27 (20.93%)	102 (79.06%)	129 (35.53%)	p = 0.01^*^
	Primary	4 (3.27%)	38 (31.14%)	80 (65.57%)	122 (33.60%)	
	High School	8 (8.79%)	25 (27.47%)	58 (63.73%)	91 (25.06%)	
	Intermediate school	1 (5.88%)	4 (23.52%)	12 (70.58%)	17 (4.68%)	
	Graduate	0 (0.0%)	1 (25.0%)	3 (75.0%)	4 (1.10%)	
Socioeconomic status	Upper class	1 (2.56%)	10 (25.64%)	28 (71.79%)	39 (10.74%)	p = 0.02^*^
	Upper middle class	5 (6.41%)	13 (16.66%)	60 (76.92%)	78 (21.48%)	
	Middle class	5 (5.10%)	30 (30.61%)	63 (64.28%)	98 (26.99%)	
	Lower middle class	1 (0.98%)	36 (35.29%)	65 (63.72%)	102 (28.09%)	
	Lower class	1 (2.17%)	6 (13.04%)	39 (84.78%)	46 (12.67%)	

The smoking status of patients and the duration of biomass fuel exposure showed a significant association with the presence of osteoporosis among study subjects (p-value < 0.0001) (Table 2).

**Table 2:** Association of smoking status and duration of biomass fuel exposure with osteoporosis ^*^ Statistically significant For statistical analysis, Pearson’s chi-square test was employed, with a p-value of less than 0.05 considered statistically significant.

Variable	Status	Osteoporosis		Total (n, %)	Statistical interpretation (chi-square test)
		Present (n, %)	Absent (n, %)		
Smoking status	Nonsmoker	112 (58.03%)	81 (41.97%)	193 (53.17%)	p < 0.0001^*^
	Current smoker	109 (80.15%)	27 (19.85%)	136 (37.48%)	
	Reformed smoker	34 (100.00%)	0 (0.0%)	34 (9.37%)	
Duration of biomass fuel exposure	Not exposed	21 (45.65%)	25 (54.35%)	46 (12.67%)	p < 0.0001^*^
	<10 years	10 (40.0%)	15 (60.0%)	25 (6.89%)	
	10-20 years	112 (77.78%)	32 (22.22%)	144 (39.67%)	
	21-40 years	93 (75.0%)	31 (25.0%)	124 (34.16%)	
	>40 years	19 (79.17%)	5 (20.83%)	24 (66.11%)	

In this study, the presence of metabolic syndrome was significantly associated with the severity of airflow limitation (p = 0.001), BMI (p < 0.001), route of corticosteroid administration (p < 0.0001), duration of corticosteroid use (p < 0.0001), education status (p = 0.006), socioeconomic status (p = 0.001), smoking status (p = 0.02), and duration of biomass fuel exposure (p = 0.008) (Table 3).

**Table 3:** Association between GOLD grading of airflow limitation severity of COPD, BMI, route of administration of corticosteroid, duration of corticosteroid used, education status, socioeconomic status of the patients, smoking status, and duration of biomass fuel exposure versus presence of metabolic syndrome ^* ^Statistically significant For statistical analysis, Pearson’s chi-square test was employed, with a p-value of less than 0.05 considered statistically significant. COPD, chronic obstructive pulmonary disease; GOLD, Global Initiative for Chronic Obstructive Lung Disease

Variable	Status	Metabolic syndrome		Total (n, %)	Statistical interpretation (chi-square test)
		Present (n, %)	Absent (n, %)		
GOLD grading of airflow limitation severity	GOLD 1	44 (68.8%)	20 (31.3%)	64 (17.6%)	p = 0.001^*^
	GOLD 2	110 (67.1%)	54 (32.9%)	164 (45.2%)	
	GOLD 3	60 (54.1%)	51 (45.9%)	111 (30.6%)	
	GOLD 4	13 (54.2%)	11 (45.8%)	24 (6.6%)	
BMI of patients	Underweight	15 (22.7%)	51 (77.27%)	66 (18.2%)	p < 0.001^*^
	Normal weight	124 (60.49%)	81 (39.51%)	205 (56.5%)	
	Overweight	79 (95.18%)	4 (4.82%)	83 (22.9%)	
	Obese	9 (100%)	0 (0.0%)	9 (2.5%)	
Route of administration of corticosteroid	Not used	48 (76.19%)	15 (23.81%)	63 (17.35%)	p < 0.0001^*^
	Oral	86 (76.11%)	27 (23.89%)	113 (31.12%)	
	Inhaled	93 (49.73%)	94 (50.27%)	187 (51.52%)	
Duration of corticosteroid used	Not used	48 (76.19%)	15 (23.81%)	63 (17.35%)	p < 0.0001^*^
	<1 year	154 (81.05%)	36 (18.95%)	190 (52.34%)	
	≥1 year	25 (22.73%)	85 (77.27%)	110 (30.30%)	
Education status	Illiterate	40 (64.51%)	22 (35.48%)	62 (17.07%)	p = 0.006^*^
	Primary	65 (67.70%)	31 (32.29%)	96 (26.44%)	
	High school	33 (66.0%)	17 (34.0%)	50 (13.77%)	
	Intermediate school	52 (71.23%)	21 (28.76%)	73 (20.11%)	
	Graduate	37 (45.12%)	45 (54.87%)	82 (22.58%)	
Socioeconomic status	Upper class	50 (75.75%)	16 (24.24%)	66 (18.18%)	p = 0.001^*^
	Upper middle class	55 (76.38%)	17 (23.61%)	72 (19.83%)	
	Middle class	36 (56.25%)	28 (43.75%)	64 (17.63%)	
	Lower middle class	41 (53.94%)	35 (46.05%)	76 (20.93%)	
	Lower class	45 (52.94%)	40 (47.05%)	85 (23.41%)	
Smoking status	Nonsmoker	106 (54.92%)	87 (45.08%)	193 (53.17%)	p = 0.02^*^
	Current smoker	92 (67.65%)	44 (32.35%)	136 (37.48%)	
	Reformed smoker	29 (85.29%)	5 (14.71%)	34 (9.37%)	
Duration of biomass fuel exposure	Not exposed	26 (56.52%)	20 (43.48%)	46 (12.67%)	p = 0.008*
	<10 years	13 (52.0%)	12 (48.0%)	25 (6.89%)	
	10-20 years	96 (66.67%)	48 (33.33%)	144 (39.67%)	
	21-40 years	70 (56.45%)	54 (43.54%)	124 (34.16%)	
	>40 years	22 (91.67%)	2 (8.33%)	24 (66.11%)	

In this study, a significant association was found between the duration of biomass fuel exposure, duration and type of tobacco smoke exposure, and BMI of the patients with different grades of GOLD airflow limitation (p-value <0.001) (Table 4).

**Table 4:** Association of duration of biomass fuel exposure, duration of tobacco smoke exposure, type of tobacco smoke exposure, and BMI status with severity of airflow limitation of COPD (GOLD grading) ^*^ Statistically significant For statistical analysis, Pearson’s chi-square test was employed, with a p-value of less than 0.05 considered statistically significant. COPD, chronic obstructive pulmonary disease; GOLD, Global Initiative for Chronic Obstructive Lung Disease

Variable	Status	GOLD airflow limitation grading				Total (n, %)	Statistical interpretation (chi-square test)
		GOLD 1 (n, %)	GOLD 2 (n, %)	GOLD 3 (n, %)	GOLD 4 (n, %)		
Duration of exposure to biomass fuel	Not exposed	28 (7.7%)	17 (4.7%)	0 (0.0%)	1 (0.3%)	46 (12.7%)	p < 0.001^*^
	<10 years	3 (0.8%)	16 (4.4%)	6 (1.7%)	0 (0.0%)	25 (6.9%)	
	10-20 years	23 (6.3%)	68 (18.7%)	49 (13.5%)	4 (1.1%)	144 (39.4%)	
	21-40 years	10 (2.8%)	46 (12.7%)	49 (13.5%)	19 (5.2%)	124 (34.1%)	
	>40 years	0 (0.0%)	17 (4.7%)	7 (1.9%)	0 (0.0%)	24 (6.6%)	
Duration of exposure to tobacco smoke	Not exposed	53 (14.6%)	114 (31.4%)	25 (6.9%)	1 (0.3%)	193 (53.2%)	p < 0.001^*^
	<10 years	4 (1.1%)	30 (8.3%)	37 (10.2%)	2 (0.6%)	73 (20.1%)	
	10-20 years	6 (1.7%)	16 (4.4%)	46 (12.7%)	15 (4.1%)	83 (22.9%)	
	21-40 years	1 (0.3%)	4 (1.1%)	3 (0.8%)	6 (1.7%)	14 (3.9%)	
Type of exposure to tobacco smoke	Not exposed	53 (14.6%)	114 (31.4%)	25 (6.9%)	1 (0.3%)	193 (53.2%)	p < 0.001^*^
	Beedi	8 (2.2%)	42 (11.6%)	62 (17.1%)	21 (5.8%)	133 (36.6%)	
	Cigarette	2 (0.6%)	2 (0.6%)	11 (3.0%)	2 (0.6%)	17 (4.7%)	
	Hookah	1 (0.3%)	6 (1.7%)	13 (3.6%)	0 (0.0%)	20 (5.5%)	
BMI of patients	Underweight	6 (9.09%)	29 (43.9%)	22 (33.3%)	9 (13.6%)	66 (18.18%)	p < 0.001^*^
	Normal weight	32 (15.6%)	99 (48.3%)	66 (32.2%)	8 (3.9%)	205 (56.47%)	
	Overweight	26 (31.3%)	29 (34.9%)	23 (27.71%)	5 (6.02%)	83 (22.86%)	
	Obese	0 (0.0%)	7 (77.7%)	0 (0.0%)	2 (22.2%)	9 (0.02%)	

Among male study subjects, the presence of metabolic syndrome was significantly associated with osteoporosis (p-value = 0.0001) (Table 5).

**Table 5:** Association of metabolic syndrome with osteoporosis among male study subjects ^*^ Statistically significant For statistical analysis, Pearson’s chi-square test was employed, with a p-value of less than 0.05 considered statistically significant.

Metabolic syndrome in male patients	Osteoporosis in male patients		Total (n, %)	Statistical interpretation (chi-square test)
	Present (n, %)	Absent (n, %)		
Present	59 (59.0%)	41 (41.0%)	100 (65.36%)	p = 0.0001^*^
Absent	48 (90.56%)	5 (9.43%)	53 (34.64%)	

Metabolic syndrome was also significantly associated with the presence of osteoporosis among female COPD patients (p-value < 0.0001) (Table 6).

**Table 6:** Association of metabolic syndrome with osteoporosis among female study subjects ^*^ Statistically significant For statistical analysis, Pearson’s chi-square test was employed, with a p-value of less than 0.05 considered statistically significant.

Metabolic syndrome in female patients	Osteoporosis in female patients		Total (n, %)	Statistical interpretation (chi-square test)
	Present (n, %)	Absent (n, %)		
Present	104 (81.89%)	23 (18.11%)	127 (60.48%)	p < 0.0001^*^
Absent	44 (53.01%)	39 (46.99%)	83 (39.52%)	

## Discussion

In this study, a total of 363 COPD patients were included, comprising 153 males and 210 females. The mean age of patients diagnosed with metabolic syndrome was 64.46 ± 10.37 years, which was slightly higher than the mean age of 57.29 ± 8.97 years reported in the study by Pasha et al. [11]. Similarly, the mean age of patients diagnosed with osteoporosis was 68.42 ± 11.38 years, also higher than the 56.04 ± 7.14 years reported in a study conducted in Egypt [12]. A significant difference in mean age was observed between patient groups with and without osteoporosis, whereas no such difference was found between those with and without metabolic syndrome. This finding is consistent with research by Cebron Lipovec et al. [4], which also reported no significant association between age and metabolic syndrome.

The severity of airflow limitation, classified using GOLD grading, showed a significant association with metabolic syndrome and T-score categories of BMD measurements. Notably, the highest number of patients diagnosed with both osteoporosis and metabolic syndrome were in GOLD grade 2 of airflow limitation severity, aligning with findings from studies by Pasha et al. [11] and EL-Gazzar et al. [12]. This can be attributed to the fact that patients with severe and very severe airflow limitation (GOLD grade 4) often experienced hemodynamic instability and additional lung infections, leading to their exclusion from the study. Meanwhile, patients with mild airflow limitation (GOLD grade 1) were often in a pre-symptomatic stage and did not seek medical attention.

The prevalence of osteoporosis and metabolic syndrome in this study was 70.25% and 62.53%, respectively. Osteoporosis was more common among female COPD patients (58.03%) than males, and similarly, metabolic syndrome was also more prevalent in females (55.95%). However, studies conducted in Karnataka [11] and Egypt [12] reported a higher prevalence of metabolic syndrome and osteoporosis, respectively, among males, in contrast to the findings of the present study. Among the 363 patients, 26.17% were diagnosed with osteopenia, while only 3.58% had normal bone density based on their DEXA scans.

In this study, BMI had a significant association with T-score categories of BMD and the presence of metabolic syndrome. A significant difference in BMI was observed between patients with and without metabolic syndrome (p < 0.0001), consistent with the findings of Cebron Lipovec et al. [4]. Additionally, BMI showed a significant association with GOLD grading of airflow limitation severity, aligning with the study by EL-Gazzar et al. [12]. The poor nutritional status of COPD patients with higher GOLD grades likely explains the link between BMI and osteoporosis. In some COPD patients, respiratory dysfunction leads to physical inactivity and reduced exercise capacity, contributing to central obesity and metabolic syndrome. This study also found significant differences between patients with and without metabolic syndrome regarding mean height (p < 0.0001), mean body weight (p = 0.001), and mean waist circumference (p = 0.001). However, no significant differences were observed between osteoporotic and non-osteoporotic patients in terms of mean height (p = 0.23) and mean body weight (p = 0.12). These findings align with Moayyeri et al. [13], who reported height loss as a predictor of fractures in COPD patients with osteoporosis. In this study, mean height, mean body weight, and mean BMI values were lower in the osteoporotic group than in the non-osteoporotic group.

A study by Pasha et al. [11] explained that both smoking and biomass fuel exposure can trigger a local inflammatory response, contributing to COPD comorbidities such as osteoporosis and metabolic syndrome. Similarly, in the present study, a significant association was found between smoking status and the presence of metabolic syndrome (p = 0.02) and osteoporosis (p < 0.0001). The duration of biomass fuel exposure among COPD patients also showed a significant association with both conditions.

Among the 363 COPD patients in this study, 53.17% were nonsmokers, 37.46% were current smokers, and 9.37% were former smokers. In comparison, Pasha et al. [11] reported that 30.3% of their study population were current smokers, while 69.7% were ex-smokers. Additionally, biomass fuel exposure, tobacco smoke exposure, and the type of tobacco used were significantly associated with GOLD severity grading of airflow limitation in the present study.

This study also found significant differences between patients with and without metabolic syndrome concerning mean systolic blood pressure, mean diastolic blood pressure, mean FBS levels, and mean serum HDL-C levels. In contrast, Pasha et al. [11] reported no significant differences in FBS levels or hypertension between cases and controls. However, consistent with the present study, they found a significant difference in HDL-C levels between these groups.

The duration and route of administration of corticosteroid-containing drugs showed a significant association with the presence of metabolic syndrome in this study. A majority of patients were unaware of the adverse effects of corticosteroids and had used them irrationally to manage disease symptoms. This lack of awareness may explain the elevated FBS levels and high prevalence of hypertension and metabolic syndrome among the study subjects. Similarly, Watz et al. [14] also highlighted the impact of corticosteroids on metabolic syndrome.

However, in this study, no association was found between the duration and route of corticosteroid use and the presence of osteoporosis. This finding aligns with the study by Jørgensen and Schwarz [15], which suggested that corticosteroid use alone cannot account for the increased prevalence of osteoporosis, as other contributing factors also play a role.

Furthermore, a significant association was observed between socioeconomic status, educational qualification, and the presence of both metabolic syndrome and osteoporosis. Most patients in this study belonged to the illiterate category and the lower-middle-income socioeconomic class. This may contribute to the irrational use of corticosteroids, often prescribed by unqualified local practitioners, as well as delayed or inadequate healthcare-seeking behavior.

Hypoxia plays a crucial role in the pathogenesis of osteoporosis by inhibiting osteoblast growth and stimulating osteoclast activity. Similarly, a lower blood pH inhibits mineral deposition by osteoblasts while activating osteoclasts, and reduced pO₂ has a similar effect [16]. Consistent with the findings of Arnett [16], the present study observed significant differences in the mean values of pH, pO₂, and SpO₂ between patients with and without osteoporosis. However, no significant difference was found in the mean partial pressure of carbon dioxide (pCO₂) between these groups.

Hypoxia also promotes adipose tissue inflammation, which disrupts insulin signaling and contributes to the development of metabolic syndrome among COPD patients [17]. Similar to the findings of Summers et al. [17], this study identified significant differences in the mean values of pO₂, SpO₂, and pCO₂ between patients with and without metabolic syndrome.

Additionally, a significant difference was found in the mean T-score values from DEXA scans and serum calcium levels between patients with and without osteoporosis, aligning with the findings of Jørgensen and Schwarz [15].

Consistent with a study conducted in Spain [18], the present study also established a significant association between metabolic syndrome and osteoporosis in both male and female COPD patients. This relationship can be explained by the fact that osteoporosis leads to reduced physical activity, which contributes to obesity - one of the key components of metabolic syndrome.

Limitations

The present study has certain limitations. Since it was not a case-control study, the exact role of COPD in the pathogenesis of osteoporosis and metabolic syndrome could not be determined. Additionally, as a cross-sectional study, it does not establish causal relationships with clinical outcomes. Furthermore, being a single-center study with a limited sample size, the findings may not be generalizable to a larger population.

## Conclusions

Osteoporosis and metabolic syndrome are highly prevalent among COPD patients in rural India, with a higher incidence in females than males. Several factors, including the severity of airflow limitation, BMI, educational status, poverty, and duration of biomass fuel exposure, significantly influence the development of both conditions. Additionally, metabolic syndrome itself contributes to the occurrence of osteoporosis in both male and female COPD patients. Those with a higher BMI, increased waist circumference, hypertension, elevated FBS levels, hypoxemia, and acidemia are at a greater risk of developing metabolic syndrome. Similarly, advanced age, hypoxemia, and acidemia play a crucial role in osteoporosis progression among COPD patients. Early detection of COPD, improved literacy, enhanced socioeconomic status, awareness of the disease and its comorbid conditions, strict regulations on the purchase of corticosteroid-containing drugs without a certified doctor’s prescription, lifestyle modifications, behavioral changes, tobacco smoking cessation, and the substitution of biomass fuel can all help reduce the prevalence of osteoporosis and metabolic syndrome in India.
